# Comparison of a Clinic-Based ELISA Test Kit with the Immunofluorescence Antibody Test for Assaying *Leishmania infantum* Antibodies in Dogs

**DOI:** 10.1155/2013/249010

**Published:** 2013-09-26

**Authors:** Daniela Proverbio, Eva Spada, Luciana Baggiani, Giada Bagnagatti De Giorgi, Roberta Perego

**Affiliations:** Dipartimento di Scienze Veterinarie per la Salute, la Produzione Animale e la Sicurezza Alimentare, Università degli Studi di Milano, Via G. Celoria, 10-20133 Milano, Italy

## Abstract

This study compares a rapid Immunospecific Kalazar Canine Rapid Spot IF with the gold standard test (indirect fluorescent antibody test (IFAT)) for detection of *Leishmania infantum* specific IgG serum antibodies in naturally exposed dogs. Serum samples were obtained from 89 healthy dogs and dogs affected by canine leishmaniosis (CanL). IgG-IFAT titers ≥80 were considered positive. Anti-*L. infantum* IgG antibodies were found in 54 samples with titers ranging from 1 : 80 to 1 : 5120. The performance of the rapid Immunospecific Kalazar was evaluated using a ROC curve. The area under the ROC curve of 0.957 was significantly different from 0.5 (*P* < 0.0001), and therefore it can be concluded that the rapid Immunospecific Kalazar has the ability to distinguish canine sera with and without *L. infantum* IgG. The best performance of the test was at a cutoff >0 (sensitivity 92.6%, specificity 97%). The test can be used for disease screening if the cutoff is >0 (highest sensitivity, 92.6%) and is recommended as confirmatory test for the presence of *L. infantum* IgG antibodies if the cutoff is set >2 (highest specificity, 100%).

## 1. **Introduction**

Canine leishmaniasis (CanL) due to *Leishmania infantum* infection is a life-threatening zoonotic disease with a wide distribution in four continents and is also important in nonendemic regions. In the Mediterranean basin canine leishmaniasis is widespread. The disease is present in central and southern regions of Italy, including the islands [1]. Based on results from a recent survey, leishmaniasis is now focally endemic in continental northern Italy [2–4].

Leishmaniasis has also been reported in northern regions of Europe such as Germany and the UK and in the USA and Canada [5–7]. *Leishmania infantum* is transmitted mainly when infected phlebotomine sandflies (*Phlebotomus* spp. and *Lutzomyia* spp. in the old and new world, resp.) [8] feed, and dogs are the main reservoir for human leishmaniasis [3, 9]. 

The diagnosis of CanL infection is complicated by nonspecific clinical presentations and variable laboratory findings. Clinical presentations range from subclinical/asymptomatic to full-blown disease, depending on the host's immune response [10]. 

The diagnosis of CanL can be made by direct methods such as cytological examination of samples from lymph nodes, bone marrow, spleen, or skin, polymerase chain reaction (PCR) on biological tissue or indirect methods for detection of anti-*Leishmania* antibodies of which the immunofluorescence antibody test (IFAT) and enzyme-linked immunosorbent assay (ELISA) are the most commonly used techniques [9, 11–13]. High antibody levels are associated with high parasitism [10] and provide a definitive diagnosis of CanL [9]. IFAT is considered the “gold standard” method for serological diagnosis of CanL with a specificity of 100% for antibody titers ≥1 : 160 [10, 12]. 

Immunochromatography tests have been developed to provide a more rapid and easy to use diagnostic test, which would also be valuable in mass screening. An immunochromatographic test for leishmaniasis antibody based on recombinant K39 (rK39), a protein predominant in *Leishmania infantum* and *Leishmania donovani* tissue amastigotes, has been developed for research purposes in both human and veterinary medicine [14–18]. This test is easy to use and provides qualitative results on the spot. In previous canine studies [14–18] this immunochromatography kit has been shown to have variable specificity (reported specificity from 61 to 100%) and sensitivity, and its performance is still not optimal [16–18].

The aim of this study was to investigate the accuracy of the results provided by this immunochromatographic test and to assess its sensitivity and specificity to measure *L. infantum* IgG antibodies in dogs, using a ROC curve and IFAT test as gold standard.

## 2. Materials and Methods

### 2.1. Canine Population and Samples

In order to reflect the situations encountered in veterinary practice, the study population was selected to have a variety of anamnestic responses and different concentrations of serum antibodies against *Leishmania infantum*. The aim was to study the test on a population similar to that on which veterinarians in practice would use the test. Nonfasting blood samples were obtained from 89 canine patients, between 2 and 14 years of age, referred to the Veterinary Clinic, Veterinary Faculty, Milan University. Fifty-four dogs had originated in, or had travelled to areas where canine leishmaniasis was endemic, and of these, 40 showed signs compatible with CanL and 14 had no clinical signs nor hematological abnormalities (anemia, thrombocytopenia), hyperproteinemia, hypergammaglobulinemia, and decreased albumin to globulin ratio (A/G ratio) compatible with CanL. In all 54 dogs, *L. infantum* infection was confirmed by either a positive real-time PCR during which *L. infantum* DNA was amplified from 200 *μ*L of whole blood using the 92 Illustra blood genomicPrep. Mini Spin kit (GE Healthcare, Milan, Italy) following the manufacturer's instructions [19] or cytological examination and a positive test for detection of *L. infantum *IgG antibodies. The control group comprised 25 clinically healthy dogs living in nonendemic areas that had never travelled to an endemic area, were treated annually to control filariasis, and were negative for *L. infantum* infection on both PCR and IFAT tests. In order to evaluate any possible cross-reaction with *Ehrlichia canis* seropositivity, a further control group of 10 dogs was included; these were from nonendemic areas for leishmaniasis and were negative on PCR and IFAT for *L. infantum* but positive on an IFAT test for detection of *E. canis *IgG antibodies. All serum samples were analyzed in a double-blind procedure to compare the ability of an in-clinic standardized immunochromatographic technique (Kalazar Canine Rapid Test, Immunospec Corporation Canoga Park, CA) to measure canine *L. infantum *IgG antibodies with the standard IFAT test. 

### 2.2. Immunochromatographic Test

The immunochromatographic Kalazar Canine Rapid Test is a qualitative membrane-based immunoassay for the detection of anti-*Leishmania* antibodies in canine serum. The membrane is precoated with rK39-antigen on the test line region and chicken antiprotein A on the control region. The test was performed according to the manufacturer's instructions. In brief the procedure was as follows: after allowing the serum specimen and the strip to reach room temperature, 20 *μ*L of serum was added to the test strip in the area beneath the arrow, the strip was then placed vertically in a test tube. Two drops of the chase buffer solution provided in the kit were added to the test tube. The results were read after 10 min. 

The test was considered positive if 2 distinct red lines appeared, one on the test region (regardless of the shade of color (red or pink)) and the other on the control region. Negative test results were reported when no line appeared in the test region, and the test was considered invalid if no line appeared in either the test or control region. The manufacturer's guide for interpretation of results reports that the intensity of the red color in the test region varies depending on the concentration of anti-*Leishmania* antibodies present, so the intensity of the red line was scored from 1 to 5, as follows: negative: no line, + (result line much paler than control line), ++ (result line paler than control line), +++ (result line equal in intensity to the control line), ++++ (result line darker than control line), and +++++ (result line much darker than control line).

### 2.3. Indirect Immunofluorescence Antibody Test 

The IFAT test was carried out as previously described [20] using a commercial kit *Leishmania*-Spot IF (BioMérieux Marcy L'Etoile, France) that uses *L. infantum* promastigotes as antigen. Briefly the parasitic cells were exposed to serum diluted in phosphate-buffered saline pH 7.2, in a damp chamber, washed, and exposed to fluorescein labeled rabbit anti-dog IgG (Sigma Aldrich, Munich Germany) at 37° for 30 minutes in a similar incubation. The slides were then washed, dried, and examined under a fluorescent microscope. Positive and negative controls were included in each series of analyzed samples. For the IFAT test cytoplasmic or membrane fluorescence at an antibody titer of 1 : 80 was considered positive as indicated by manufacturer's instructions.

The IFAT test for detection of *E. canis *IgG antibodies test was performed using a commercial kit, Fluo *Ehrlichia canis* (Agrolabo). Serum samples were titrated using 10 serial twofold dilutions in PBS, starting at a 1 : 40 dilution. Diluted serum (10 *μ*L) was placed into each well of a 12-well antigen-coated slide containing cultured cells infected with *E. canis* as antigens. A positive control serum and PBS as a negative control were also placed in separate wells. After incubation at 37°C for 30 min, the slide was washed three times with PBS. Then one drop of fluorescein isothiocyanate-conjugated goat anti-dog, immunoglobulin G, was placed into each well, and the plates were incubated at 37°C in the dark and rinsed as described above. A coverslip was placed on the slide with mounting medium, and the slide was examined under an epifluorescent microscope. An antibody titer of 1 : 80 was considered positive.

To establish the repeatability of the immunochromatographic test, 4 canine samples with IFAT titers of negative, 1 : 80, 1 : 160, and 1 : 320, were analyzed 10 times on the same day. 

Conservability was tested by performing the immunochromatographic test on 2 samples (IFAT titer 1 : 80 and 1 : 320, resp.) stored at −20°C for 24 and 48 hours and for 7, 15, and 30 days, respectively.

### 2.4. Statistical Analysis

A weighted-*k* statistic (*k*) with 95% confidence interval was calculated to evaluate agreement greater than chance alone between the 2 testing methods, comparing between the titer of positivity of the IFAT and the degree of positivity detected by immunochromatographic test expressed in 5 grades of color intensity. The level of agreement between the 2 testing methods, based on *k*, was scored according to the following guidelines: 0: no better than chance; 0.20: <poor agreement; 0.21–0.40: fair agreement; 0.41–0.60: moderate agreement; 0.61–0.80: good agreement; 0.81–1.00: very good agreement [21].

Data obtained from the immunochromatographic Kalazar Canine Rapid Test were checked for normal distribution using the Kolmogorov-Smirnov test, while the difference between the positive and negative groups was calculated using the Kruskal-Wallis test. The diagnostic agreement between the two variables obtained by IFAT and Kalazar was determined using Spearman's coefficient of rank correlation. To assess overall performance of the immunochromatographic test, sensitivity, specificity, negative (LR−) and positive (LR+) likelihood ratios, negative predictive value (PV−), and positive predictive value (PV+) were calculated generating a ROC curve using IFAT test as criterion-reference standard [22]. The performance of the test was analyzed by comparing the area under the curve (AUC), 1 indicating a perfect test and 0.5 indicating results similar to chance. The area under the ROC curve provides a single numerical estimate of overall accuracy that can be interpreted as the average probability that an infected animal will have a positive test value compared to a noninfected animal.

When the variable being considered cannot distinguish between the group of negative and positive, that is, where there is no difference between the two distributions, the AUC will be equal to 0.5, and the ROC curve will coincide with the diagonal. When there is a perfect separation of the values of the two groups; that is, the distributions do not overlap, the area under the ROC curve equals 1.

All statistic analyses were performed using MedCalc for Windows v. 9.2.1. (Mariakerke, Belgium). For all analyses, values of *P* < 0.05 were considered significant. 

## 3. Results

Anti-*L. infantum* IgG antibodies were found in 54/89 samples using the IFAT test, with titers ranging between 1 : 80 and 1 : 5120, and 51/89 samples were positive with the rapid immunochromatographic test. Five samples gave discordant results: one IFAT negative sample tested positive with the rapid test and 4 positive IFAT samples tested negative with immunochromatography (Table 1). Weighted k statistics comparing agreement by the 2 methods in detecting the level of anti-*Leishmania* antibodies were 0.228 demonstrating fair agreement beyond chance.

**Figure 1:**
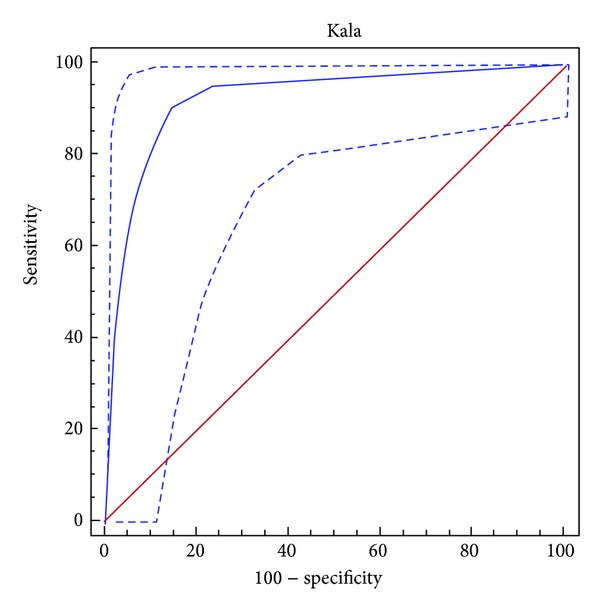
ROC curve for the immunochromatographic assay for detection of anti-*Leishmania* IgG antibodies. *Y*-axis shows the false positive rate (specificity), and the *x*-axis shows the true positive rate (sensitivity). A test with the perfect discrimination has a ROC curve that passes through the upper left corner. The area under the curve (AUC) is *W* = 0.957 (*P* > 0.0001). The ROC analysis shows that the cutoff point for the test with the best sensitivity/specificity is >0 (Se: 92.1%; Sp: 97.14% expressed as percentages).

Data obtained by the immunochromatographic Kalazar Canine Rapid Test were not normally distributed (Kolmogorov-Smirnov, *P* < 0.001) with significant differences between the groups of samples that tested positive and negative for anti-*Leishmania* IgG antibodies (Kruskas-Wallis, *P* < 0.0001). Spearman's coefficient of rank correlation was 0.832 (95% CI: 0.754–0.886). Assessment of the ROC curve showed an AUC (*W* = 0.957) relative to the Immunochromatographic test. This is very close to an AUC of 1 (which indicates a perfect test) and significantly greater (*P* < 0.0001) than the AUC that characterizes a test unable to discriminate serum with and without *L. infantum*-specific IgG (*W* = 0.5) (Figure 1). The ROC analysis showed that the test cutoff point with the best sensitivity/specificity is >0 Se (92.1%; 95% CI: 82.1%–97.9%) and Sp (97.14%; 95% CI: 85%–99.5%), +LR 32.41 (95% CI: 4.7–223.1%), −LR 0.08 (95% CI: 0.08–0,2%), PPV di 98 (95% CI: 88.2–99.8%) and NPV 89.4 (95% CI: 74.2–96.5%). Neither the IFAT *L. infantum* test nor the immunochromatographic test was positive for anti-*Leishmania* IgG antibodies in the 10 samples that were positive for *E. canis* antibodies. 

The repeatability of the immunochromatographic assay was good. The same results were recorded in all 10 tests repeated on the 3 samples with variation in the degree of positivity in only 3 out of 10 repeated tests on the 1 : 80 titer sample (Table 2). The immunochromatographic assay also returned the same result in tests performed after the different storage periods. 

## 4. Discussion

Canine leishmaniasis represents not only a serious veterinary disease but also a public health problem, since dogs are the main reservoir of the parasite and play a key role in the transmission cycle. Early serological diagnosis is essential to confirm disease and indicate a requirement for start therapy; for screening clinically healthy dogs who live in or originate from endemic regions; for the detection of subclinical carriers in blood donor pools and to investigate the presence of infection in epidemiological studies for surveillance control programmes [13]. 

The most useful diagnostic approaches for detection of infection in both sick and clinically healthy infected dogs include detection of specific antileishmanial serum antibodies by several serological techniques and demonstration of the parasite DNA in tissues by molecular techniques. The most accessible tests for the detection of specific serum antibodies (IgG) are quantitative serological techniques, such as the immunofluorescence antibody test (IFAT) and enzyme-linked immunosorbent assay (ELISA) [23]. These tests, however, are time-consuming and require technological expertise and specialized laboratory equipment.

Notwithstanding the great number of techniques and protocols currently available, many diagnostic challenges remain especially for the practitioner, and a single easy to use and reliable tool for disease diagnosis would be invaluable. 

Immunochromatographic-based assays are easy to use and rapid and provide qualitative results on the spot. They require neither special preparation of the sample nor special equipment, and the execution times are quick (10 minutes). Additionally the Kalazar Canine Rapid Test may be stored at ambient temperature and is suitable for field use.

Immunochromatographic test for the rapid detection of anti-*L. infantum* IgG is able to distinguish canine serum samples with and without these antibodies. The best sensitivity and specificity of the test occur for values greater than zero that is a positive test at the lowest color intensity. The Se and Sp of a test vary according to the cutoff value chosen, and this may change depending on the purpose for which the test is being used. The most important requirement when using a test to screen for infection is sensitivity [24]. The results of this study suggest that the immunochromatographic test is useful as a rapid screening test for the presence of *L. infantum*-specific serum antibodies in dogs when a cutoff value ≥0 is used (Se 92.59%; NPV 89.5%). At this cutoff, the rapid Kalazar Canine Rapid Test could be a useful screening method in epidemiological studies of the seroprevalence of canine leishmaniasis. Although in endemic areas and for surveillance programs sensitivity is essential, in clinical cases specificity is more important [22]. Our results suggest that the rapid immunochromatographic test can be used to confirm the presence of *L. infantum*-specific IgG, provided that the cutoff value for identification of clinically suspected animals is very positive, that is, grade 2. At this level both the Sp and PPV are 100%, eliminating the risk of identifying a healthy animal as infected. The weighted *k* statistic showed there was fair agreement between the 2 methods with regard to the degree of positivity. 

The poor correlation between the IFAT titers and the intensity of the positive red line on the test strip may be caused by the different antigen profiles presented in each test: whole parasites for IFAT test and recombinant antigen specific for visceral leishmaniasis for the immunochromatographic test that can determine a different affinity of antibodies. Three of four false negative results occurred in samples with a low positive titer using the IFAT test (1 : 80). The presence of low antibody level is not necessarily indicative of disease and must be evaluated in relation to clinicopathological signs and confirmed by other tests such as parasitological test [25]. Thus, in the clinical setting, negative Kalazar Canine Rapid Test results must always be considered in conjunction with clinical signs, and additional diagnostic tests will be needed for confirmation of disease in clinically suspect dogs in which the test is negative. 

## 5. Conclusions

In conclusion, our study shows that the Kalazar Canine Rapid Test is useful in the clinical setting to distinguish dogs with or without *L. infantum*-specific IgG if a positive result is defined as grade 2 (the color of the red result line being slightly paler than control line), whilst exclusion of false positives can only be achieved if a higher level of specificity is demanded from the test. 

Perfect correlation between the degrees of agglutination of the 2 methods would have allowed their interchangeable use in clinical practice. On the basis of our results we suggest that the Kalazar Canine Rapid Test is only used to confirm disease in dogs in which there is clinical suspicion of disease. Alternative methods, such as ELISA or IFAT, are needed to provide a qualitative measurement of the serum antibody concentration and an endpoint titer.

## Figures and Tables

**Table 1:** Distribution of 89 canine serum samples according to the different antibody titers identified with IFAT and immunochromatographic assay for the detection of anti-*Leishmania* IgG antibodies.

IgG Immunochromatographic test	IgG IFAT								
	<1 : 80	1 : 80	1 : 160	1 : 320	1 : 640	1 : 1280	1 : 2560	1 : 5120	Total
Result and degree of positivity									
−	34	3	1	0	0	0	0	0	38
+	0	4	2	0	0	0	0	0	6
++	1	1	2	0	0	0	2	0	6
+++	0	2	0	3	0	0	0	2	7
++++	0	2	3	3	1	2	2	1	14
+++++	0	1	3	4	4	3	2	1	18

Total	35	13	11	10	5	5	6	4	89

**Table 2:** Repeatability of the immunochromatographic assay for the detection of IgG anti-*Leishmania* antibodies: results obtained by repeating the immunochromatographic test 10 times on the same sample, in four different samples, with different titles of positivity (negative, 1 : 80, 1 : 160, and 1 : 320 according to the IFAT test). The red line was more intense in 3 out of 10 repeated tests on the sample with a titer of 1 : 80.

IFAT test	1	2	3	4	5	6	7	8	9	10
Neg	−	−	−	−	−	−	−	−	−	−
1 : 80	++++	**+++++**	++++	++++	++++	**+++++**	**+++++**	++++	++++	++++
1 : 160	++++	++++	++++	++++	++++	++++	++++	++++	++++	++++
1 : 320	+++++	+++++	+++++	+++++	+++++	+++++	+++++	+++++	+++++	+++++

## References

[1] Gramiccia M (2011). Recent advances in leishmaniosis in pet animals: epidemiology, diagnostics and anti-vectorial prophylaxis. *Veterinary Parasitology*.

[2] Maroli M, Rossi L, Baldelli R (2008). The northward spread of leishmaniasis in Italy: evidence from retrospective and ongoing studies on the canine reservoir and phlebotomine vectors. *Tropical Medicine and International Health*.

[3] Biglino A, Bolla C, Concialdi E, Trisciuoglio A, Romano A, Ferroglio E (2010). Asymptomatic Leishmania infantum infection in an area of Northwestern Italy (Piedmont region) where such infections are traditionally nonendemic. *Journal of Clinical Microbiology*.

[4] Spada E, Proverbio D Canine leishmaniasis in non-endemic area: incidence of positivity to IFAT test in dogs moved to endemic area.

[5] Gothe R, Nolte I, Kraft W (1997). Leishmaniosis of dogs in Germany: epidemiological case analysis and alternative to conventional causal therapy. *Tierarztliche Praxis*.

[6] Duprey ZH, Steurer FJ, Rooney JA (2006). Canine visceral leishmaniasis, United States and Canada, 2000–2003. *Emerging Infectious Diseases*.

[7] Shaw SE, Langton DA, Hillman TJ (2009). Canine leishmaniosis in the United Kingdom: a zoonotic disease waiting for a vector?. *Veterinary Parasitology*.

[8] Dante-Torres F, Solano-Gallego L, Baneth G, Marcio RV, Cavalcanti MP, Otranto D (2012). Canine leishmaniosis in the old and new words: unveiled similarities and difference. *Trends in Parasitology*.

[9] Solano-Gallego L, Koutinas A, Miró G (2009). Directions for the diagnosis, clinical staging, treatment and prevention of canine leishmaniosis. *Veterinary Parasitology*.

[10] Reis AB, Martins-Filho OA, Teixeira-Carvalho A (2009). Systemic and compartmentalized immune response in canine visceral leishmaniasis. *Veterinary Immunology and Immunopathology*.

[11] Alvar J, Cañavate C, Molina R, Moreno J, Nieto J (2004). Canine leishmaniasis. *Advances in Parasitology*.

[12] Maia C, Campino L (2008). Methods for diagnosis of canine leishmaniasis and immune response to infection. *Veterinary Parasitology*.

[13] Miró G, Cardoso L, Pennisi MG, Oliva G, Baneth G (2008). Canine leishmaniosis—new concepts and insights on an expanding zoonosis: part two. *Trends in Parasitology*.

[14] Iqbal J, Hira PR, Saroj G (2002). Imported visceral leishmaniasis: diagnostic dilemmas and comparative analysis of three assays. *Journal of Clinical Microbiology*.

[15] Reithinger R, Quinnell RJ, Alexander B, Davies CR (2002). Rapid detection of Leishmania infantum infection in dogs: comparative study using an immunochromatographic dipstick test, enzyme-linked immunosorbent assay, and PCR. *Journal of Clinical Microbiology*.

[16] Otranto D, Paradies P, Sasanelli M (2005). Recombinant K39 dipstick immunochromatographic test: a new tool for the serodiagnosis of canine leishmaniasis. *Journal of Veterinary Diagnostic Investigation*.

[17] Mettler M, Grimm F, Capelli G, Camp H, Deplazes P (2005). Evaluation of enzyme-linked immunosorbent assays, an immunofluorescent-antibody test, and two rapid tests (immunochromatographic-dipstick and gel tests) for serological diagnosis of symptomatic and asymptomatic Leishmania infections in dogs. *Journal of Clinical Microbiology*.

[18] Quinnell RJ, Carson C, Reithinger R, Garcez LM, Courtenay O (2013). Evaluation of rK39 rapid diagnostic tests for canine visceral leishmaniasis: longitudinal study and meta-analysis. *PLOS Neglected Tropical Diseases*.

[19] Reale S, Maxia L, Vitale F, Glorioso NS, Caracappa S, Vesco G (1999). Detection of Leishmania infantum in dogs by PCR with lymph node aspirates and blood. *Journal of Clinical Microbiology*.

[20] Altman DG (1991). *Practical Statistics for Medical Research*.

[21] Mancianti F, Meciani N (1988). Specific serodiagnosis of canine leishmaniasis by indirect immunofluorescence, indirect hemagglutination, and counterimmunoelectrophoresis. *American Journal of Veterinary Research*.

[22] Gardner IA, Greiner M (2006). Receiver-operating characteristic curves and likelihood ratios: improvements over traditional methods for the evaluation and application of veterinary clinical pathology tests. *Veterinary Clinical Pathology*.

[23] Ferroglio E, Vitale F (2006). Diagnosis of leishmaniosis: between old doubts and new uncertainties. *Veterinary Research Communications*.

[24] Lappin MR, Greene CE, Prestwood AK, Dawe DL, Marks A (1989). Prevalence of *Toxoplasma gondii* infection in cats in Georgia using enzyme-linked immunosorbent assays for IgM, IgG, and antigens. *Veterinary Parasitology*.

[25] Ferroglio E, Centaro E, Mignone W, Trisciuoglio A (2007). Evaluation of an ELISA rapid device for the serological diagnosis of Leishmania infantum infection in dog as compared with immunofluorescence assay and Western blot. *Veterinary Parasitology*.

